# Impact of Invisalign G-series Updates on Improving Predicted Outcomes: A Retrospective Study

**DOI:** 10.7759/cureus.50615

**Published:** 2023-12-16

**Authors:** Eman Fatani, Hadeel B Alkhamsi, Faisal O Arishi, Shoug M Altaweel, Malak A Asiri, Walaa W Albuni, Mohammad A Baseer

**Affiliations:** 1 Preventive Dentistry, College of Medicine and Dentistry, Riyadh Elm University, Riyadh, SAU; 2 Dentistry, College of Medicine and Dentistry, Riyadh Elm University, Riyadh, SAU; 3 Dentistry, College of Dentistry, Prince Sattam bin Abdulaziz University, Al-Kharj, SAU; 4 Dentistry, College of Dentistry, Majmaah University, Al Majma'ah, SAU; 5 Dentistry, College of Dentistry, King Khalid University, Abha, SAU; 6 Dentistry, Vision Colleges, Riyadh, SAU

**Keywords:** percentage accuracy, orthodontic treatment, achieved outcome, predicted outcome, invisalign g-series

## Abstract

Background: Understanding the real-world implications of periodic changes to orthodontic appliances can provide valuable insights for future treatment strategies and patient outcomes. This study aimed to investigate the impact of the latest updates added to the G7 and G8 Invisalign series on actual versus predicted outcomes and the percentage accuracy of the treatment.

Method: This retrospective study was conducted in private orthodontic practices in Riyadh, Saudi Arabia. Orthodontists carried out Invisalign® treatment using the latest updates added to the G7 and G8 Invisalign series. The study group comprised patients with different malocclusion types who received non-extraction Invisalign treatment. The Invisalign treatment plan was provided by the ClinChecks program (Invisalign, San Jose, United States) for patients treated throughout the years (2016-2022). Different dimensions were assessed to record predicted and actual treatment outcomes with the aid of iTero® (Align Technology, San Jose, United States) and ClinCheck® (Invisalign, San Jose, United States). The percentage accuracy was determined using the formula (100%-((Predicted-Actual)/Predicted) *100%).

Results: A total of 108 patients (male = 34 (31.5%) and female = 74 (68.5%)) treated with Invisalign G7 and G8 series were considered in this study. The overall mean and standard deviation values of vertical distance (2.91±1.42), intermolar distance in the lower arch (52.68±3.15), overjet (2.71±1.06), and inter-incisal angle (138.24±12.18) were higher than the predicted model. However, the predicted model showed higher mean and standard deviation values for intercanine distances in the upper (36.94±1.57) and lower arches (28.48±1.40) and upper intermolar distances (57.21±2.91). The G7 versus G8 intercanine distance in lower (61.28±47.67 vs. 80.51±38.32), intermolar distance in upper (61.72±47.67 vs. 69.95±44.11), and intermolar distance in lower (100.68±3.80 vs. 100.89±2.52) were relatively higher in the G8 series than the G7. The accuracy percentage was higher with the G8 series than with the G7 regarding the intercanine distance in the upper arch. In contrast, the G7 series showed a higher mean percentage accuracy of vertical distance (91.11±84.83 vs. 76.76±65.45), overjet (58.44±35.17 vs. 53.71±45.87), and inter-incisal angle (34.47±44.06 vs. 27.53±37.98) than the G8 series.

Conclusion: The percentage accuracy of aligner therapy administered using the Invisalign G7 and G8 series demonstrated no significant variation.

## Introduction

The inception of clear aligner treatment marked a significant shift in orthodontic therapeutics in the late 20th century. This innovative technique, brought to life by Align Technology, introduced a transparent aligner for dental alignment, providing a revolutionary alternative to traditional alignment methods [1,2]. The Invisalign appliance unveiled in 1999, offered a solution for mild malocclusions, addressing the rising demand among adults for a way to improve their smiles without resorting to braces.

Align Technology (San Jose, United States) has since made strides in enhancing its technology, refining its 3D interface, integrating diverse attachment designs to assist various types of tooth movement, and upgrading its software to calibrate precise force determinations for each tooth movement, thereby allowing for the treatment of a wide array of malocclusions [3].

The iTero Element scanner (Align Technology, San Jose, United States), another innovative tool in orthodontics, demonstrated increased speed and negligible errors, thus providing an economic advantage for practitioners. However, it was observed that the scanning accuracy was somewhat lower in the upper jaw compared to the lower jaw [4-7]. Introduced by Cadent LTD and later reimagined by Align Technology in 2013, the iTero® scanner was developed to work in synergy with the OrthoCad® and Invisalign Clincheck® software (both produced by Align Technology, San Jose, United States). This integration enables clinicians to review and adjust the proposed treatment plans when utilizing Invisalign® technology [8,9]. In 2013, Diamond Braces (Englewood, United States) introduced the SmartTrack material, a multi-layer polymer, replacing the standard aligner material. This innovation significantly reduced the peak intensity and duration of pain, as well as insertion pressure [10]. Align Technology has progressively updated its G-series software, incorporating clinical innovations to improve treatment outcomes. This evolution began with G3 in 2010, followed by G4 in 2011, G5 in 2014, and the most recent update, G8, with smart force aligner activation, introduced in 2021 [11].

Earlier studies have used 3-dimensional (3D) superimposition of predicted treatment outcomes onto the actual models as a technique to assess Invisalign's accuracy in predicting various types of tooth movements. However, it was reported that multi-trial digital superimpositions were repeatable with reduced errors. The average accuracy of anterior tooth movement with Invisalign was 41% [11,12], while canine rotation and incisor intrusion were the least accurately predicted movements. On the other hand, all horizontal and extrusion movements of the incisors were found to be the most accurate [13]. Tien et al. reported mean accuracy rates of 72.2% and 82.3% for upper and lower intercanine widths, respectively [14], while upper and lower intermolar widths showed accuracy rates of 63.5% and 79.8%. Recent reports indicated that Invisalign predicted an overall mean accuracy of 76.85% [15].

It's important to note that most of these studies are based on the Invisalign G7 series. Invisalign G8 introduces a series of enhancements designed to improve the predictability of deep-bite correction. Among these advancements is the implementation of balanced anterior en-masse intrusion, a technique that ensures uniform movement of anterior teeth. The G8 system also incorporates a newly optimized attachment specifically designed for the lower lateral incisor. This innovation enhances the grip and control during the movement of these teeth, thereby increasing the predictability of the treatment outcome. Another significant modification in the G8 version is the overcorrection of lower incisor intrusion and the flattening of the Curve of Spee. Overcorrection is a strategy implemented to account for potential relapse, while the flattened Curve of Spee helps in achieving better occlusion and function. There's a need for more research to increase the predictability of treatment since predicted tooth movements do not completely coincide with realized tooth movements. Hence, this study aimed to investigate the impact of the latest updates added to the G7 and G8 Invisalign series on actual versus predicted outcomes and the percentage accuracy of treatment. The null hypothesis examined in this research states that there is no significant difference in the percentage accuracy of aligner therapy administered using the Invisalign G7 and G8 series.

## Materials and methods

Ethical approval

This retrospective cohort study was authorized by the Institutional Review Board of Riyadh Elm University and assigned the approval number SRP/2021/87/505/484.

Study sample

The samples used in this study were selected at the investigators' discretion and were designated as convenience samples. The information was obtained from private orthodontic practices in Riyadh, Saudi Arabia, where orthodontists were all Invisalign® Diamond providers or above who carried out treatment regimens. The study group comprised patients with different malocclusion types who received non-extraction Invisalign® treatment, including all its variants and improvements, resulting in the submission of at least two approved ClinChecks®. Wearing aligners lasts three weeks, with an average treatment time of one year. The Invisalign treatment plan was provided by the ClinChecks program for patients treated throughout the years (2016-2022). For each patient, we created a digital treatment plan using a unique program called ClinCheck. We were provided with the anticipated treatment result at the end of this strategy. We employed a technology that captures a 3D scan of the patient's mouth to see the actual outcome of the treatment. We were able to compare the expected and real results using iTero Element. We superimposed the digital models from ClinCheck onto the iTero Element's 3D images using OrthoCAD, another program. By using this comparison, we were able to quantify a number of tooth motions, including the angle formed by the upper and lower front teeth, the amount of overlap between them, and the widening of the space between the canines and molars. Only patient records that satisfied our particular requirements were included. Records that did not fit these requirements were not included in the analysis.

Selection criteria

The study included patients who were healthy and had been exclusively treated with Invisalign. It was necessary for these patients to demonstrate good compliance during their therapy. They had to have undergone both an initial and a concluding intraoral digital scan. Furthermore, only patients who had used at least one refinement set were included. On the other hand, individuals were excluded from the study if they presented with systemic disease, syndromes, or if they had a cleft lip and palate. Non-compliance with aligner wear also resulted in exclusion. Patients who had undergone dental procedures or oral surgery prior to the final scan were not included in the study. The study also excluded any patient under the age of 18 and those with any missing teeth, with the exception of the third molars.

The patient files were selected from the ClinChecks program in conformity with the inclusion criteria, while the age was determined by subtracting the current year from the patient's birthdate. The data and scans were immediately linked to the ClinChecks program and an iTero. The scanner was used to capture and superimpose the first refinement scan from the patient's record with the first scan of the initial treatment in the system. The software for tracking progress and the report are regarded as Align's confidential information and cannot be given to any third party. Two digital models were presented. The models can be viewed and evaluated in four planes, with one representing the initial dentition scan and the other representing the dentition for its most recent scans (sagittal, vertical, transverse, and arch length). The stages of each aligner were recorded independently to compare the predictable outcome of ClinChecks with the first refinement scan. The progress assessment was divided into four categories: initial measurement, current measurement, programmed measurement, and final measurement. In the context of this study, four postgraduate students who were selected for this investigation were significant in both the management of samples and data analysis. In a setting like ours, postgraduate students were involved in all stages of the research process under the supervision and guidance of three experienced orthodontists. This included the collection and management of samples, conducting the analyses, interpreting the results, and contributing to the writing of the final report or study. In terms of quality control and supervision, experienced professionals and faculty advisors oversaw the entire process to ensure the accuracy and reliability of the data analysis. They ensured that the research methodologies were correctly applied, data analysis was conducted appropriately, and the results were accurate and reliable.

Statistical analysis

During this phase, an array of statistical procedures were executed to discern the relationship between anticipated and actual outcomes. Specifically, both descriptive and inferential statistical methodologies were employed to evaluate the variables of interest. Initially, separate computations of the central tendency (mean) and dispersion (standard deviation) were accomplished for the G7 and G8 series of Invisalign treatments. These calculations facilitated an understanding of the typical behavior of these variables and the extent of their deviation. As a subsequent step, we performed a normality test - a statistical process used to determine if a dataset is well-modeled by a normal distribution. By assessing the skewness and kurtosis of our data set, we gauged its approximation to a Gaussian distribution. To quantify the discrepancies between expected and observed results for the G7 and G8 series, we employed the independent samples t-test. This inferential statistical test allowed us to analyze whether the mean difference between the two groups (expected and actual outcomes) was statistically significant. To measure the degree of linear correlation between predicted and actual variables, we utilized Pearson's correlation coefficient. A strong positive correlation would imply a high degree of predictability between the expected and actual outcomes, while a weak or negative correlation would suggest less predictability or an inverse relationship. To verify the precision of our findings, we juxtaposed the actual results against the predicted outcomes. The accuracy of the results was determined by the degree of congruence between these two data sets. These calculations were executed utilizing the statistical software IBM SPSS Statistics for Windows, Version 25 (Released 2017; IBM Corp., Armonk, New York, United States). Statistical significance was inferred if the p-value was less than 0.05, suggesting that the observed results would be highly unlikely under the null hypothesis.

## Results

A total of 108 patients (male=34 (31.5%) and female=74 (68.5%)) treated with Invisalign G7 and G8 series were considered in this study. Table 1 shows the distribution of patients treated with the G7 and G8 Invisalign series. Most patients had class I malocclusion 72 (66.7%), with 21 patients treated with G7 and 51 treated with the G8 Invisalign series. Twenty-three patients with class II malocclusion were treated with the G7 (n = 6) and G8 (n = 17) Invisalign series. Only 13 patients with class III malocclusion were treated with the G7 (n = 7) and G8 (n = 8) series (Table 1).

**Table 1:** Distribution of patients treated with G7 and G8 Invisalign series

Variable analyzed	G7		G8		Total	
	n	%	n	%	n	%
Gender						
Male	14	41.2%	20	27.0%	34	31.5%
Female	20	58.8%	54	73.0%	74	68.5%
Total	34	100.0%	74	100.0%	108	100.0%
Classification						
Class I	21	61.8%	51	68.9%	72	66.7%
Class II	6	17.6%	17	23.0%	23	21.3%
Class III	7	20.6%	6	8.1%	13	12.0%
Total	34	100.0%	74	100.0%	108	100.0%

The overall mean and standard deviation values of vertical distance (2.91±1.42), intermolar distance in the lower arch (52.68±3.15), overjet (2.71±1.06), and inter-incisal angle (138.24±12.18) were higher than the predicted model. However, the predicted model showed higher mean and standard deviation values for intercanine distances in the upper (36.94±1.57) and lower arches (28.48±1.40) and upper intermolar distances (57.21±2.91). The mean difference between predicted and achieved measures differed significantly in vertical dimension, upper intercanine and intermolar distances, overjet, and inter-incisal angle (p<0.05). All the studied variables showed a significant positive correlation between predicted and achieved measurements (p<0.05) except for the lower intermolar distance (r=-0.092, p=0.345). The highest mean percentage accuracy was observed with lower intermolar distance measurement (100.82±2.96), and the lowest percentage accuracy was found with inter-incisal angle (29.72±39.91), as shown in Table 2.

**Table 2:** Overall mean±SD, correlation, and percentage accuracy of predicted and achieved variables * means statistical significance at the 0.05 level and ** denotes statistical significance at the 0.01 level

Variables	Predicted (mm/degrees)		Achieved (mm/degrees)		Predicted and achieved (mm/degrees)		Accuracy (%)		Difference (mm/degrees)		
	Mean	SD	Mean	SD	Correlation	p-value	Mean	SD	Mean	SD	95% CI
Vertical	1.90	0.70	2.91	1.42	.525^**^	<0.001	81.28	72.02	-1.002	1.212	-1.233 - -0.771*
Intercanine (upper)	36.94	1.57	36.74	1.66	.915^**^	<0.001	74.46	42.23	0.201	0.671	0.073 - 0.329*
Intercanine (lower)	28.48	1.40	28.40	1.44	.871^**^	<0.001	67.36	45.20	0.074	0.720	-0.063 - 0.211
Intermolar (upper)	57.21	2.91	56.22	3.20	.857^**^	<0.001	73.30	42.20	0.982	1.660	0.666 -1.299*
Intermolar (lower)	47.46	48.34	52.68	3.15	-0.092	0.345	100.82	2.96	-5.220	48.728	-14.515 - 4.075
Overjet	2.47	0.65	2.71	1.06	.325^**^	0.001	55.20	42.68	-0.242	1.045	-0.441 - -0.042*
Inter-incisal angle	135.58	7.25	138.24	12.18	.637^**^	<0.001	29.72	39.91	-2.656	9.397	-4.449 - -0.864*

**Figure 1:**
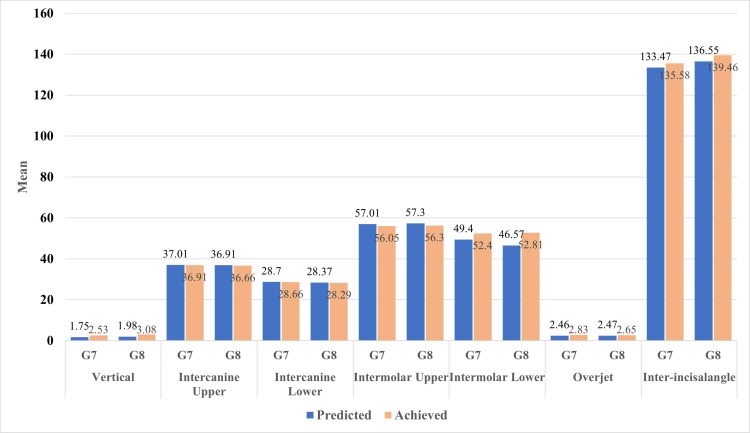
Graphical representation of correlation and percentage accuracy of predicted and achieved variables of G7 and G8 Invisalign series

The mean±SD, correlation, and percentage accuracy of predicted and achieved variables of the G7 and G8 Invisalign series are shown in Figure 1 and Table 3, respectively. The G7 series demonstrated higher achieved mean and standard deviation values for vertical distance (2.53±1.33), intermolar distance in the lower arch (52.40±2.77), overjet (2.83±0.95), and inter-incisal angle (135.58±10.35) than the predicted model. However, the mean difference between predicted and achieved models was significant for vertical dimension and overjet measures (p<0.05). On the other hand, the G7 series showed higher predicted values of upper intercanine distance (37.01±1.45), lower intercanine distance (28.70±1.46), and upper intermolar distance (57.01±2.38) than the achieved models. The mean difference between the predicted and achieved models was statistically significant in the upper intermolar distance. In addition, all the studied variables measured using the G7 series demonstrated a statistically significant correlation between predicted and achieved models (p<0.05). The G7 series showed the highest mean percentage accuracy of (100.68±3.80) with the lower intermolar distance, and the lowest mean percentage accuracy of (34.47±44.06) was observed with inter-incisal angle measurement.

**Table 3:** Mean±SD, correlation, and percentage accuracy of predicted and achieved variables of G7 and G8 Invisalign series * means statistical significance at the 0.05 level and ** denotes statistical significance at the 0.01 level

Measurements	Gen	Predicted (mm/degrees)		Achieved (mm/degrees)		Predicted and achieved (mm/degrees)		Percentage accuracy (mm/degrees)		Difference (mm/degrees)		
		Mean	SD	Mean	SD	Correlation	p-value	Mean	SD	Mean	SD	95% CI
Vertical	G7	1.75	0.80	2.53	1.33	0.494	0.003	91.11	84.83	-0.779	1.166	-1.186 - -0.373*
	G8	1.98	0.63	3.08	1.44	0.530	<0.001	76.76	65.45	-1.104	1.226	-1.388 - -0.820*
Intercanine (upper)	G7	37.01	1.45	36.91	1.66	0.851	<0.001	61.28	47.67	0.103	0.873	-0.202 - 0.407
	G8	36.91	1.63	36.66	1.67	0.943	<0.001	80.51	38.32	0.246	0.555	0.117 - 0.375*
Intercanine (lower)	G7	28.70	1.46	28.66	1.61	0.868	<0.001	61.72	47.67	0.041	0.802	-0.239 - 0.321
	G8	28.37	1.37	28.29	1.34	0.873	<0.001	69.95	44.11	0.089	0.685	-0.069 - 0.248
Intermolar (upper)	G7	57.01	2.38	56.05	2.82	0.786	<0.001	68.75	44.56	0.959	1.753	0.347 - 1.570*
	G8	57.3	3.14	56.3	3.37	0.878	<0.001	75.39	41.21	0.993	1.627	0.616 - 1.370*
Intermolar (lower)	G7	49.40	48.26	52.40	2.77	-0.420	0.013	100.68	3.80	-2.997	49.485	-20.263 -14.269
	G8	46.57	48.68	52.81	3.32	0.033	0.783	100.89	2.52	-6.242	48.682	-17.521- 5.037
Overjet	G7	2.46	0.79	2.83	0.95	0.539	0.001	58.44	35.17	-0.365	0.844	-0.659 - -0.070*
	G8	2.47	0.58	2.65	1.11	0.227	0.051	53.71	45.87	-0.185	1.126	-0.446 - 0.076
Inter-incisal angle	G7	133.47	7.52	135.58	10.35	0.475	0.005	34.47	44.06	-2.109	9.471	-5.413 - 1.196
	G8	136.55	6.96	139.46	12.81	0.695	<0.001	27.53	37.98	-2.908	9.417	-5.090 - -0.726*

Similarly, the G8 series demonstrated higher achieved mean and standard deviation values for vertical distance (3.08±1.44), lower intermolar distance (52.81±3.32), overjet (2.65±1.11), and inter-incisal angle (139.46±12.81) than the predicted model. However, the mean difference between predicted and achieved models was significant for vertical dimension and inter-incisal angle measures (p<0.05). Contrarily, the G8 series demonstrated higher predicted than achieved mean and standard deviation values for upper intercanine (36.91±1.63), lower intercanine (28.37±1.37), and upper intermolar distance measurements (57.3±3.14). The mean difference between the predicted and achieved models was statistically significant in upper intercanine and upper intermolar distances (p<0.05). Moreover, all the studied variables demonstrated a statistically significant correlation between predicted and achieved models (p<0.05) except for lower intermolar and overjet measures. The G8 series showed the highest mean percentage accuracy of (100.89±2.52) with the lower intermolar distance, and the lowest mean percentage accuracy of (27.53±37.98) was observed with the inter-incisal angle measurement.

**Figure 2:**
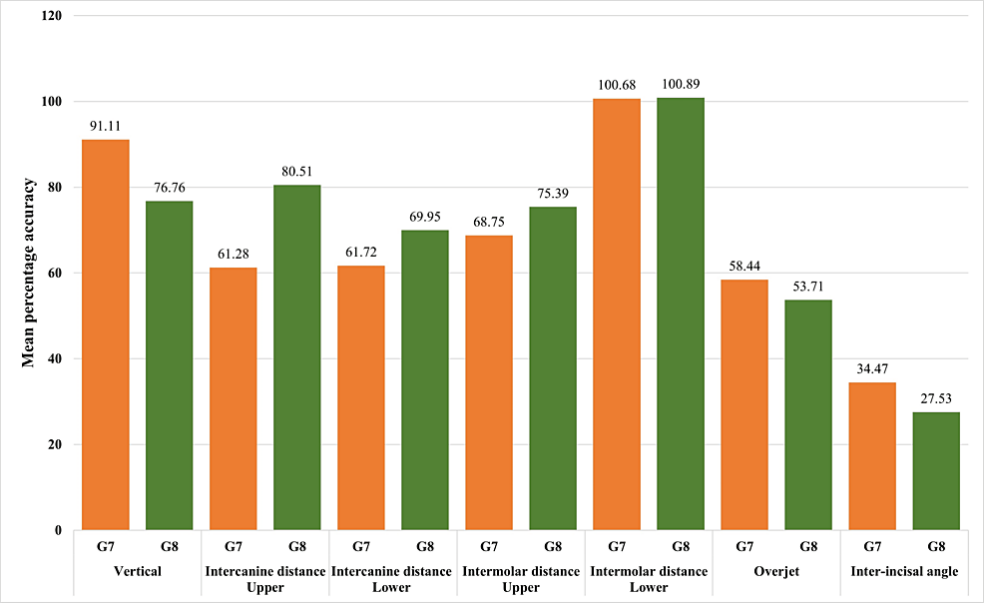
Graphical representation of the percentage accuracy of variables between G7 and G8 Invisalign series

The comparison of the mean percentage accuracy of variables measured between the G7 and G8 Invisalign series is shown in Figure 2 and Table 4, respectively. The G7 versus G8 intercanine distance in lower (61.28±47.67 vs. 80.51±38.32), intermolar distance in upper (61.72±47.67 vs. 69.95±44.11), and intermolar distance in lower (100.68±3.80 vs. 100.89±2.52) were relatively higher in the G8 series than the G7. The accuracy percentage was significantly higher with the G8 series than with the G7 regarding the intercanine distance in the upper arch. In contrast, the G7 series showed a higher mean percentage accuracy of vertical distance (91.11±84.83 vs. 76.76±65.45), overjet (58.44±35.17 vs. 53.71±45.87), and inter-incisal angle (34.47±44.06 vs. 27.53±37.98) than the G8 series.

**Table 4:** Comparison of percentage accuracy of variables between G7 and G8 Invisalign series

Variables	Series	N	Mean (mm/degrees)	SD (mm/degrees)	SEM	t	p
Vertical	G7	34	91.11	84.83	14.55	0.962	0.338
	G8	74	76.76	65.45	7.61		
Intercanine distance (upper)	G7	34	61.28	47.67	8.18	-2.065	0.044
	G8	74	80.51	38.32	4.46		
Intercanine distance (lower)	G7	34	61.72	47.67	8.18	-0.877	0.382
	G8	74	69.95	44.11	5.13		
Intermolar distance (upper)	G7	34	68.75	44.56	7.64	-0.758	0.450
	G8	74	75.39	41.21	4.79		
Intermolar distance (lower)	G7	34	100.68	3.80	0.65	-0.349	0.727
	G8	74	100.89	2.52	0.29		
Overjet	G7	34	58.44	35.17	6.03	0.533	0.595
	G8	74	53.71	45.87	5.33		
Inter-incisal angle	G7	34	34.47	44.06	7.56	0.838	0.404
	G8	74	27.53	37.98	4.41		

## Discussion

As per our findings, the specific advantages promoted by the manufacturer for the Invisalign® G8 series were not detailed. However, given the iterative nature of the Invisalign versions, it can be assumed that the G8 series was designed with enhancements aimed at improving treatment predictability and outcome accuracy over its predecessors. As for the study's validation of these assumed claims, it's important to note that the findings you provided indicate that both the G7 and G8 series showed statistically significant differences between predicted and achieved values for multiple variables. For the G7 series, the achieved values were higher than predicted for vertical distance, intermolar distance in the lower arch, overjet, and inter-incisal angle. The mean differences between predicted and achieved values were significant for vertical dimension and overjet measures. On the other hand, the G7 series showed higher predicted values than achieved for upper intercanine distance, lower intercanine distance, and upper intermolar distance, with a significant mean difference in the upper intermolar distance. Turning to the G8 series, the observed mean and standard deviation values for vertical distance, lower intermolar distance, overjet, and inter-incisal angle were higher than the predicted model. The predicted model showed higher mean values for the upper and lower intercanine distances and upper intermolar distances. The mean difference between predicted and achieved measures was significantly different in several areas, including vertical dimension, upper intercanine and intermolar distances, overjet, and inter-incisal angle.

Although several authors have studied the effectiveness of Invisalign, the impact of the Invisalign G series on predictable outcomes and percentage accuracy still needs to be addressed. The average accuracy with clear aligners ranged from 55% to 72% [16]. In this study, we retrospectively evaluated the difference between the G7 and G8 regarding the predictable outcome of Invisalign treatment. Most patients had class I malocclusion (66.7%), 21.3% had class II malocclusion, and only 12.0% had class III malocclusion. Invisalign® recommends using sagittal correctors before beginning aligner therapy for severe class II malocclusions [17,18] irrespective of the need for extractions.

However, G8 demonstrated slightly superior accuracy to G7 in measuring distances between canine and molar teeth. In terms of upper jaw measurements, G8 displayed approximately 81% accuracy compared to G7's 61% for intercanine distance. Conversely, G7 surpassed G8 in vertical distance accuracy, showing about 91% accuracy as opposed to G8's roughly 77%. Furthermore, G7 demonstrated superior accuracy in measurements of overjet and interincisal angle. Our retrospective study further yielded an unexpected result: the accuracy for intermolar distance in the lower jaw exceeded 100% in both G7 and G8. In accordance with our findings, Grünheid et al. [19] reported approximately 88% accuracy for lower jaw expansion.

Invisalign's accuracy for upper jaw expansion was around 73%, although more tipping was observed than predicted in the digital treatment plan, a phenomenon also noted by Haouili et al. [20]. The average accuracy of maxillary arch transverse expansion was 70%, irrespective of the type of tooth, as reported by Galluccio et al. [21].

In the context of the broader research, the accuracy of predicted outcomes has been a crucial area of investigation. Alswajy et al. [15] conducted a comprehensive study focusing on assessing the accuracy of ClinCheck® in measuring various dental dimensions, including sagittal, vertical, transverse, and arch length dimensions. Their study reported a high correlation between the predicted and achieved outcomes for the upper intercanine width, showcasing an impressive accuracy of 97.97% with a mean difference of 0.53±1.05 mm. The results of our retrospective study align with these findings, demonstrating that the Invisalign G8 series also exhibits a high accuracy in the upper intercanine measurements (80.51±38.32), albeit less than that reported by Alswajy et al. The mean difference observed between predicted and achieved outcomes in our study was 0.246±0.555 for the G8 series, slightly lower than reported by Alswajy et al [15]. Similarly, the G7 series showed a mean difference of 0.103±0.873 in the upper intercanine measurements, indicating the G-series' ability to predict outcomes with reasonable accuracy. However, there is also evidence of discrepancies between predicted and achieved outcomes. For instance, Krieger et al. [22] reported a slight negative difference (-0.13±0.59) between the achieved and predicted tooth movement in the upper intercanine distance, and a positive difference (0.13±0.59) in the lower intercanine distance. This variation underscores that while Invisalign treatments can predict outcomes with a high degree of accuracy there may still be slight variations in the final results depending on individual patient factors and treatment complexities. Taken together, these studies contribute to our understanding of the predictive accuracy of Invisalign treatments and the potential for slight variations between predicted and achieved outcomes. These findings can be instrumental for practitioners in managing patient expectations and optimizing treatment planning.

As far as the literature is concerned in this regard, the field of Invisalign research is dynamic and diverse, with different studies focusing on various aspects of treatment outcomes. For instance, Charalampakis et al. [13] emphasized that changes in the distance between upper canine teeth were the most noticeable, attributing this to the fact that these teeth have the longest roots. Contrarily, our findings spotlighted the most significant difference in the lower molar distance, with a difference of -2.997±49.485 in G7 and -6.242±48.682 in G8 between the expected and actual outcomes. This divergence from Charalampakis et al.'s findings highlights the complexity of predicting outcomes in Invisalign treatment and suggests that factors other than root length may impact tooth movement. In terms of overjet accuracy, our findings echoed those of Krieger et al. [22] and Alswajy et al. [15]. In G7, the overjet was 58.44% accurate, with a difference of -0.365 between expected and actual outcomes, similar to the findings of Krieger et al. In the G8 series, the accuracy was slightly lower at 53.71%, with a mean difference of -0.185, aligning with Alswajy et al.'s findings. This suggests a relatively consistent degree of predictability across different series of Invisalign aligners. However, there are nuances to consider. Buschang et al. [23] and Tsai et al. [24] found that the actual overjet was slightly higher than predicted, indicating that the control over overjet and tooth tilt might be reduced when certain features are used. This underscores the need for detailed patient-specific treatment planning and highlights the importance of considering the potential influence of specific features on treatment outcomes.

In terms of vertical movement (the up-down direction), the G7 series was more accurate than the G8, with accuracies of 91.11% and 76.76%, respectively. Krieger et al. [22] have reported that vertical movement with Invisalign is more difficult, resulting in larger deviations from expected outcomes. This aligns with our findings, which showed a mean difference of -0.779 between expected and actual outcomes in G7. Alswajy et al. [15] reported a similar difference in the amount of overlap of the upper and lower front teeth (overbite). However, Castroflorio et al. [25] found that the lower front teeth were more likely to move vertically than the back teeth. Ko et al. [17] also reported that vertical and side-to-side corrections may be difficult.

Several studies [2,13,19,20] have reported that the least predictable movements are in the vertical direction, especially the movement of teeth into the jawbone (intrusion). Conversely, Bilello et al. [26] found that intrusion was quite predictable. Jaber et al. [27] reported that moving teeth out of the jawbone (extrusion) is difficult with aligners and that the aligners covering the biting surfaces of the teeth can prevent the bite from settling properly.

This study found the interincisal angle to have an accuracy percentage of 34.47±44.06 and a mean difference between the predicted and achieved values of -2.109±9.471 for the G7 series. In contrast, G8 showed 27.53±37.98 percent accuracy, and the mean difference between predicted and achieved was -2.908±9.417, representing the lowest percentage of all the parameters. In contrast, Alswajy et al. [15] reported a higher percentage accuracy of 96.23% in their study.

Despite the use of various calculation formulas, there was a limitation in this study, with aberrant results in lower intermolar distance. The vertical distance, overjet, and inter-incisal angle measurements indicated that G7 is more accurate than G8, regardless of a larger sample size for G8. However, the larger sample size somewhat offset the bias that could be attributed to the findings. Furthermore, it should be noted that we only investigated the recent generation series of Invisalign (G7, G8) to determine if it has improved the actual outcome of the aligner treatment over the projected one for each plane, not per tooth for every movement. It seemed logical to develop strategies to increase the predictability of achieving the desired results after identifying the limits of clear aligner therapies. A range of conceptual approaches to improve the efficacy and efficiency of clear aligners are provided by Bowman [28].

## Conclusions

Based on the retrospective study, it was observed that the Invisalign G-Series updates had a significant impact on improving the predicted outcomes. The mean difference between the predicted and achieved measurements showed significant variations in several variables, such as vertical dimension, upper intercanine and intermolar distances, overjet, and inter-incisal angle. However, all studied variables demonstrated a significant positive correlation between predicted and achieved measurements, except for the lower intermolar distance. When comparing the G7 and G8 Invisalign series, both demonstrated higher achieved mean values for vertical distance, intermolar distance in the lower arch, overjet, and inter-incisal angle than the predicted model. However, the G8 series showed relatively higher accuracy in the intercanine distance in the lower arch, and intermolar distances in both the upper and lower arch, as compared to the G7 series. The G7 series, on the other hand, demonstrated a higher mean percentage accuracy of vertical distance, overjet, and inter-incisal angle than the G8 series. Conclusively speaking, the Invisalign G-Series updates were found to be effective in improving predicted outcomes, with some variations between the G7 and G8 series. This information may be beneficial to orthodontists since G8 series overrides the G7 series for specific orthodontic measurements and treatment outcomes. However, it is important to consider that while the series of updates shows promising results, individual patient factors and treatment complexities can influence the final outcomes.
